# Supplementary material for: Free roaming of 3D stratum models based on internal and external boundary identification
Source: PLoS One. 2024 May 31;19(5):e0300805. doi: 10.1371/journal.pone.0300805 (PMC11142589; doi:10.1371/journal.pone.0300805)
Supplement: Attachment — Submitted filename: Response to Reviewers.docx [file pone.0300805.s002.docx]

**Original Article Title: “Free roaming of 3D stratum models based on internal and external boundary identification**”

**To:** PLOS ONE Editor

**Re:** Response to reviewers

Dear Editor,

Thank you for allowing a resubmission of our manuscript, with an opportunity to address the reviewers’ comments.

We are uploading (a) our point-by-point response to the comments (below) (response to reviewers, under “Response to Reviewers*”*), (b) an updated manuscript with yellow highlighting indicating changes (as “Revised Manuscript with Track Changes*”*), and (c) a clean updated manuscript without highlights (“Manuscript”*).*

Best regards,

Yusen Zhong.

**Academic Editor, Concern # 1:** Please ensure that your manuscript meets PLOS ONE's style requirements, including those for file naming.

**Author response:** Thank you for reminding me. We have modified the format of this article according to the template you provided.

**Academic Editor, Concern # 2:** Thank you for stating the following financial disclosure:

"The research is supported by the National Natural Science Foundation of China (NSFC) (Grant Numbers: 42293354, 42293351, 42293355, 42277131, 41977230).

These NSFC fundings are all awarded by the author Cuiying Zhou.

The model software and equipment are funded by these sponsors."

Please state what role the funders took in the study. If the funders had no role, please state: "The funders had no role in study design, data collection and analysis, decision to publish, or preparation of the manuscript."

If this statement is not correct you must amend it as needed.

Please include this amended Role of Funder statement in your cover letter; we will change the online submission form on your behalf.

**Author response:** Thank you for reminding me. We have stated in the cover letter as follows” This research is supported by the National Natural Science Foundation of China (Grant Numbers: 42293354, 42293351, 42293355, 42277131, 41977230). These fundings are all awarded by the corresponding author Cuiying Zhou. We declare that the funder has no known competing financial interests or personal relationships that could have appeared to influence the work reported in this paper. The funders had no role in study design, data collection and analysis, decision to publish, or preparation of the manuscript.”

**Academic Editor, Concern # 3:** In your Data Availability statement, you have not specified where the minimal data set underlying the results described in your manuscript can be found. PLOS defines a study's minimal data set as the underlying data used to reach the conclusions drawn in the manuscript and any additional data required to replicate the reported study findings in their entirety. All PLOS journals require that the minimal data set be made fully available. For more information about our data policy, please see http://journals.plos.org/plosone/s/data-availability.

**Author response:** Thank you for reminding me. Relevant data supporting the results of this study have been uploaded to a file named " stratum modeling data".

**Academic Editor, Concern # 4:** We note that you have stated that you will provide repository information for your data at acceptance. Should your manuscript be accepted for publication, we will hold it until you provide the relevant accession numbers or DOIs necessary to access your data. If you wish to make changes to your Data Availability statement, please describe these changes in your cover letter and we will update your Data Availability statement to reflect the information you provide.

**Author response:** Thank you for reminding me.

Relevant data supporting the results of this study have been uploaded to a file named " stratum modeling data".

**Academic Editor, Concern # 5:** Please amend the manuscript submission data (via Edit Submission) to include author Yusen Zhong.

**Author response:** Thank you for your reminder. We have revised the submission data.

**Academic Editor, Concern #6:** Please ensure that you refer to Figure 10 in your text as, if accepted, production will need this reference to link the reader to the figure.

**Author response:** Thank you for your reminder. We refer to Figure 10 on line 271 of this paper.

**Academic Editor, Concern # 7:** We note you have included a table to which you do not refer in the text of your manuscript. Please ensure that you refer to Table 1 in your text; if accepted, production will need this reference to link the reader to the Table.

**Author response:** Thank you for your reminder. We refer to Table 1 on line 414 of this paper.

**Reviewer#1, Concern # 1:** It is recommended to label the meaning of the rectangle in Fig. 2, and Fig. 4 as well.

**Author response:** Thank you for your advice. Figure 2 and Figure 4 have been modified and we have indicated the meaning of the rectangles marked in Figure 2 and Figure 4.

**Reviewer#1, Concern # 2:** It is recommended that the dotted lines representing the planes in Figure 9 be of a different color to make the planes clearer.

**Author response:** Thank you for your advice. Figure 9 has been modified and we have used dashed lines of different colors to represent the plane of Figure 9.

**Reviewer#1, Concern # 3:** A scale is advised to be added to the vertical coordinates of Table 1.

**Author response:** Thank you for your advice. We have added the scale in Table 1.

**Reviewer#1, Concern # 4:** Numbers 1-6 in Figure 10 are supposed be annotated with their meaning in the figure..

**Author response:** Thank you for your advice. We have commented on numbers 1 through 6 in Figure 10.

**Reviewer#1, Concern # 5:** List the inadequacies of current researches and the innovations of this article point by point in the introduction. Here, the reviewer recommends the following articles for the author's references: Rapid Path Extraction and Three-Dimensional Roaming of the Virtual Endonasal Endoscope.Three-dimensional coupled hydromechanical analysis of localized joint leakage in segmental tunnel linings.

**Author response:** Thank you for your advice. First of all, we have listed the shortcomings of the current research and the innovations of this paper point by point in the introduction. Secondly, we have referred to the two articles you provided and quoted them in the article.

**Reviewer#1, Concern # 6:** The notes in Figure 11 should be in English , not in another language.

**Author response:** Thank you for reminding me and we have modified the annotations in Figure 11.

**Reviewer#1, Concern # 7:** Mention the crisp conclusions in the conclusion section. Use academic language to write conclusions.

**Author response:** Thank you for your professional comments on our article. We have rewritten the conclusion section according to your suggestion. The conclusion (1), and (2) show the contributions of this thesis and the conclusion (3) shows the limitations of this paper.

**Reviewer#2, Concern # 1:** What are the real time applications of the proposed model. Verify your model with a real time application.

**Author response:** Thank you for your question. This model is mainly applied to three-dimensional underground space roaming. Based on the 3D stratigraphic model, it is easy to analyze the internal structure of the 3D stratigraphic model by roaming inside the 3D stratigraphic model. We developed the model primarily through the 3D roaming engine (Three.js), using WebStorm. Among them, the 3D Roaming Engine (Three.js) is a toolkit for JavaScript. A brief introduction is provided on lines 98 through 112 of this paper. Based on your suggestion, a brief explanation is given in lines 310 through 312 of this paper.

**Reviewer#2, Concern # 2:** Discuss the novelty clearly in the abstract. How the devised model is performed better than the other techniques.

**Author response:** Most studies on 3D scene roaming do not consider the case of viewpoint entering into 3D model. Moreover, whether it is a hollow 3D model or a solid 3D model, as long as the viewpoint enters the interior of the 3D model, the internal structure of the model cannot be accurately displayed. However，In geotechnical and geological engineering, it is often necessary to carry out visual analysis of the interior of 3D formation models.And the technology can meet the needs of both internal and external roaming in engineering practice, effectively solving the problems faced by the internal roaming of the 3D stratum models and providing sound roaming effects. What's more, the technology uses a more concise way to switch between internal roaming and external roaming. This paper gives a brief analysis in lines 398 to 411.

**Reviewer#2, Concern # 3:** Write the motivation behind the study in the introduction part.

**Author response:** Thank you for your advice. The research motivation is added in lines 93 to 97 of this paper.

**Reviewer#2, Concern # 4:** Write the paper structure at the end of the Introduction.

**Author response:** Thank you for your advice. We have added paper structure at the end of the introduction

**Reviewer#2, Concern # 5:** Improve the writing quality of the paper. The entire manuscript should be

proofread throughly. The grammatical and typo errors should be corrected.

**Author response:** Thank you for reminding me. We have checked the full text and made corrections where there are errors.

**Reviewer#2, Concern # 6:** A Related work section should be added. Also, discuss the research gaps of the existing methods.

**Author response:** Thank you for your advice. In lines 93 to 97 of the introduction, we add an analysis of existing research gaps.

**Reviewer#2, Concern # 7::** Provide the numbers for the equations. Define all the variables in the equations.

**Author response:** Thank you for your advice. We've defined all the variables in the equation.

**Reviewer#2, Concern # 8:** Compare your model with other related methods.

**Author response:** Thank you for pointing this out. In lines 453-455, this method is compared with existing methods and their advantages are explained.

**Reviewer#2, Concern # 9:** Discuss the limitations of your model.

**Author response:** Thank you for pointing this out. The limitations of this method are explained in lines 467-471of this paper.

**Reviewer#2, Concern # 10:** Discuss further extension of your model.

**Author response:** Thank you for pointing this out. At the end of this paper, the further extension of this model is discussed.
